# Effect of Graphene Nanosheets on the Microstructure and Mechanical Properties of Sn-20Bi Solder

**DOI:** 10.3390/ma16041550

**Published:** 2023-02-13

**Authors:** Wenchao Yang, Weiou Qin, Jingwu Wu, Junli Feng, Yongzhong Zhan

**Affiliations:** 1 School of Resources, Environment and Materials, Guangxi University, Nanning 530004, China; 2 State Key Laboratory of Featured Metal Materials and Life-Cycle Safety for Composite Structures, Nanning 530004, China; 3 The Testing and Technology Center for Industrial Products, Shenzhen Customs, Shenzhen 518067, China

**Keywords:** Sn-20Bi solder, melt-casting, fracture, intermetallic compounds, corrosion resistance

## Abstract

The application of Sn-Bi series solder is limited due to the brittleness of Bi phase. Sn-20Bi solder with less Bi element content has great research prospects, but it needs modification to make it a substitute for traditional Sn-Pb solder. In this article, we mixed graphene nanosheets with nanometer Sn powder by means of ultrasonic oscillation, and Sn-20Bi-*q*GNS (*q* = 0.01, 0.02, 0.04, 0.06, and 0.1 wt.%) solder alloys were prepared by the melt-casting method. The effects of graphene nanosheets (GNSs) on the microstructure, physical properties, mechanical properties, and corrosion resistance of solder alloys were investigated. Scanning electron microscopy, energy dispersive spectroscopy, X-ray diffraction, and X-ray photoelectron spectroscopy were used to determine the microstructural morphology and composition. The results showed that the melting point, density, and wettability of the solder decreased slightly with the addition of GNSs. The addition of GNSs as a second phase refined the solder structure and improved the tensile strength of the molten Sn-20Bi composite solder to 99.6 MPa, while elongation decreased with the addition of GNSs. Furthermore, GNSs prevented the MC Sn-20Bi-*q*GNSs/Cu intermetallic compound layers’ growth by interfering with atomic diffusion and grain boundary movement. In addition, the addition of 0.02 wt.% GNSs enhanced the shear strength of MC Sn-20Bi solder joints to 46.3 MPa. The electrochemical experimental results show that the surface corrosion products of MC Sn-20Bi-*q*GNSs under 3.5% NaCl solution were Sn_3_O(OH)_2_Cl_2_, with MC Sn-20Bi-0.01GNSs exhibiting the best corrosion resistance.

## 1. Introduction

Tin–lead solder is widely used in the electronics industry because of its low cost, good solder reliability, and excellent mechanical properties. However, due to the non-negligible risks of lead to the human body and environment, lead is required to be removed from electronic products. Currently, many lead-free solders have been studied in various fields as alternatives to Sn-Pb solders, such as Sn-Ag-Cu [1,2], Sn-Bi [3,4], Sn-Zn [5], Sn-Cu [6], Sn-In [7], and so on. Among them, the Sn-Bi series has been extensively studied by researchers for its lower cost and good wettability [8,9,10]. Sn-58Bi eutectic alloys have been widely used in industry, but the high content of Bi leads to high brittleness [11]. Therefore, reducing the mass ratio of Bi in the alloy became a solution. Lai [12,13] reported results showing that Sn-20Bi solder alloys have higher tensile strength than Sn-*x*Bi (*x* = 10, 25, 35) solder alloys. Therefore, Sn-20Bi alloys are of high research value in low Bi solder alloys.

In our previous study, Al and Cu were added to Sn-20Bi as new components. The results showed that the addition of Al reduced the wettability and improved the hardness and corrosion resistance of the alloy [14]. In addition, the combined effect of Al and Cu improved the melting properties of the alloy but reduced the electrical and mechanical properties of the alloy [15]. In addition, we added graphene nanosheets (GNSs) to the Sn-20Bi solder alloy by the sintering method [16]. It was found that a small amount of GNSs added could significantly inhibit the growth of intermetallic compound (IMC) layers at the interface and refine the microstructure. In addition, it can be concluded by nanoindentation that the addition of small amounts of GNS can reduce the hardness/elastic modulus ratio of IMC. However, due to the high preparation cost of the sintering process, we tried to prepare the Sn-20Bi-GNSs solder alloy using the less costly melt-casting (MC) method.

According to previous research, the bonding effect of graphene materials in solder alloys is poor. To ensure that graphene could be well-bonded to the metal substrate and evenly distributed, in this study, graphene nanosheets and nanotin powder were pretreated using an ultrasonic oscillation method, the powder was evenly mixed using a ball-milling method, and Sn-20Bi-GNSs filler metal was prepared using a fusion casting method. The effects of adding GNSs on the physical and mechanical properties and corrosion properties of Sn-20Bi filler metal for melt casting were studied.

## 2. Experimental

**Figure 1:**
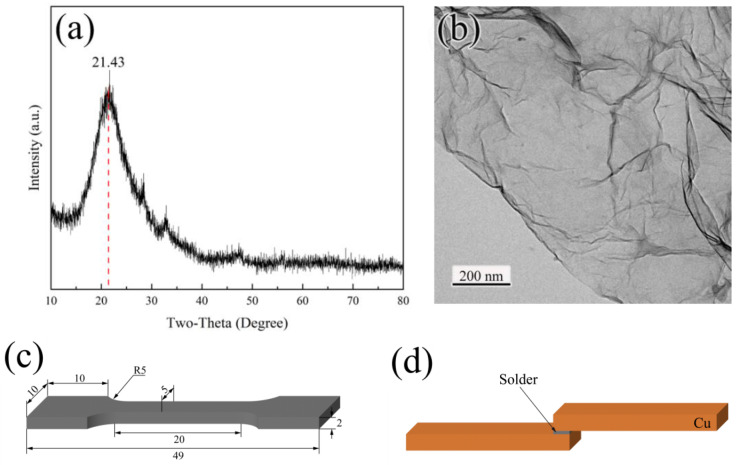
Raw materials GNSs: (**a**) XRD pattern, (**b**) TEM image, and the schematic of (**c**) tensile and (**d**) shear test samples.

In this experiment, the graphene nanosheets (Suzhou Carbon Feng Graphene Technology Co., Ltd., Suzhou, China) were prepared by the Hummer method, and their X-ray diffractometer (XRD, Rigaku D/Max 2500 V, Rigaku Corporation, Tokyo, Japan) pattern and transmission electron microscope (TEM, FEI Titan G2 ETEM, Thermo Fisher Scientific, Waltham, MA, USA) image are shown in Figure 1a,b. First, graphene nanosheets were added to ethylene glycol solution for dispersion by ultrasound. The dispersed GNSs were mixed with the Sn powder at a mass ratio of 1:200, and the mixed solution was sonicated for 60 min. The mixed solution was then extracted and dried to obtain the Sn-GNSs mixed powder. In order to make the powder mixture homogeneous, vacuum ball milling was performed. The ball milling was carried out at 250 r/min for 3 h using a 1:10 mass ratio of alloy balls to powder, and the powder was pressed into flakes at 20 MPa for 30 min to facilitate melting. Melting was carried out in a muffle furnace using the molten salt method. The composition of the molten salt was LiCl and KCl with a mass ratio of 1:3. The temperature of the muffle was kept at 400 °C. When the molten salt melted in the crucible, Sn blocks, Bi blocks, and Sn-GNSs were added to the crucible in turn for 20 min and stirred thoroughly. Finally, the liquid metal was poured into a mold and cooled in air to obtain MC Sn-20Bi-*q*GNS (*q* = 0.01, 0.02, 0.04, 0.06, and 0.1 wt.%). Image-Pro Plus 6.0 (Media Cybernetics, Inc., Montgomery, MD, USA) software was used to measure the IMC layer area at 10 different locations of 3 samples with the same composition and calculate the average thickness (thickness = area/length).

The specimens were embedded in epoxy resin and then ground and polished using standard metallographic techniques. The microstructure was observed using a scanning electron microscope (SEM, Sigma300, Cral Zeiss AG, Jena, Germany). The elemental composition of the alloy was also determined using energy dispersive spectroscopy (EDS, oxford instruments, Oxford, UK). The alloy samples were phase identified by an XRD operating at 40 kV, 200 mA, and a scanning rate of 8 °/min.

The melting characteristics of the solder alloy were measured by differential scanning calorimetry (DSC) equipment produced by Sateram Company(Bengaluru, India). The heating rate was 5 °C/ min, and the temperature was kept at 300 °C for 5 min. The solder alloy was cut into Φ6.5 mm × 1.24 mm discs, which were ground and polished to remove surface stains and tested multiple times using a high precision density meter to obtain the average density. The composite solders were put in an alumina ceramic crucible and melted into a solder ball under flux protection. To test the spreading area, the solder balls were put on a polished and cleaned 40 mm × 40 mm × 2 mm pure copper plate, reflowed at 230 °C, and air-cooled.

The solder alloy was cut into the samples shown in Figure 1c for tensile testing, which was used to test the ultimate tensile strength of the composite solder. The shear test was designed to investigate the shear strength and fracture behavior of the solder joints. The shear model was designed as shown in Figure 1d with a solder joint size of 10 mm × 5 mm × 1 mm and a copper plate size of 50 mm × 10 mm × 5 mm. The composite solder was sandwiched between two copper plates, which were fixed on a heated platform. The samples were held at 230 °C for 8 min and finally cooled to room temperature in air. Both tensile and shear tests were performed using a universal mechanical testing machine (Instron 8801, Instron, Norwood, MA, USA) to test the samples at a tensile rate of 0.6 mm/min.

The samples were cut into cylindrical shapes of 14 mm in diameter and 4 mm in thickness. The sample surfaces were ground with 1000#, 2000#, 3000#, and 5000# sandpaper and then used for electrochemical experiments. The corrosion solution used for the test was a 3.5 wt.% NaCl solution, and the surface area of the sample exposed to the test solution was 0.785 mm^2^. Three tests were performed on each sample using a CHI660 electrochemical workstation manufactured by Shanghai Chenhua Instrument Co (Shanghai, China). This workstation contains a three-electrode configuration. The reference potential was a saturated glycerol electrode and a platinum counter electrode. The scan rate was 0.002 V/s, ranging from −2.5 V to 0.5 V. SEM and EDS were used to observe the corrosion morphology of the sample surface and the composition of the surface corrosion products after polarization tests. X-ray photoelectron spectroscopy (XPS, Specs XPS/INA-X, SPECS, Berlin, Germany) was used to examine the elemental composition and valence states in the electrochemical corrosion products.

## 3. Results and Discussion

### 3.1. Phase Composition and Microstructure of Composite Solders

**Figure 2:**
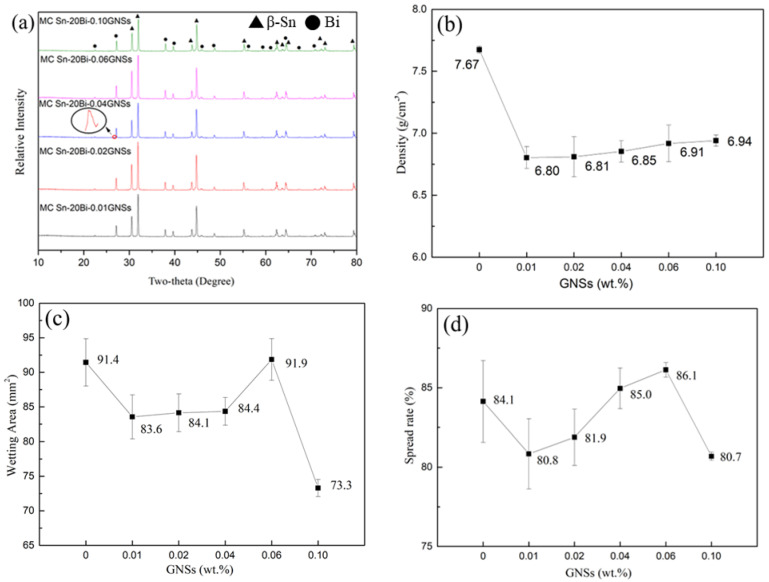

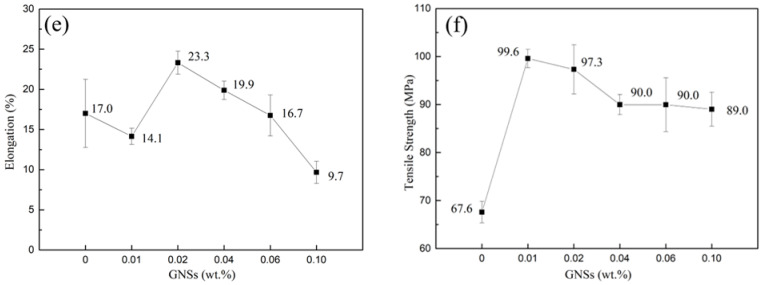
(**a**) The XRD patterns of MC Sn-20Bi-*q*GNSs (*q* = 0.01, 0.02, 0.04, 0.06, 0.10); (**b**) the density of MC Sn-20Bi-*q*GNSs; (**c**) the wetting area of MC Sn-20Bi-*q*GNSs alloys; (**d**) the spreading rate of MC Sn-20Bi-*q*GNSs alloys; (**e**) the elongation of MC Sn-20Bi-*q*GNSs alloys; and (**f**) the tensile strength of MC Sn-20Bi-*q*GNSs alloys.

The XRD patterns of MC Sn-20Bi-*q*GNSs (*q* = 0.01, 0.02, 0.04, 0.06, 0.10 wt.%) alloys prepared by the melt-casting method are shown in Figure 2a. The results show that all the MC Sn-20Bi-*q*GNSs series alloys are composed of both β-Sn and Bi phases. To further verify the effect of the addition of the GNSs, a fine sweep of MC Sn-20Bi-GNSs alloy in the range of 2θ angle from 26° to 27° (scanning speed of 0.1 °/min) was performed in this study. Starting from MC Sn-20Bi-0.04GNSs, the sample showed a small peak near 26.44°, and the peak at this position was reported as GNSs according to the literature [17]. This indicates the successful incorporation of graphene into the matrix.

**Figure 3:**
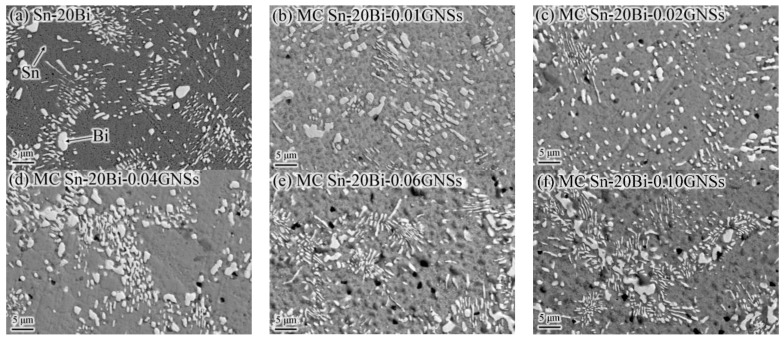
SEM images of MC Sn-20Bi-*q*GNSs alloys: (**a**) *q* = 0, (**b**) *q* = 0.01, (**c**) *q* = 0.02, (**d**) *q* = 0.04, (**e**) *q* = 0.06, and (**f**) *q* = 0.10.

The microstructure of the MC Sn-20Bi-*q*GNSs alloy is shown in Figure 3. Combined with the XRD of Figure 2a, the microstructure of all solders is a combination of white granular/elongated Bi phase and gray β-Sn substrate phase. With the addition of GNSs, a uniformly distributed dark gray dotted GNSs phase appeared in the tissue, the bias of the white Bi phase was suppressed, and the Bi phase tended to be gradually and uniformly distributed in the tissue. When the added amount of GNSs was 0.01 wt.%, the Bi particles were significantly larger and more uniformly distributed. The most uniform distribution of Bi particles was observed when the GNSs were added at 0.02 wt.%, probably because the lamellar GNSs hindered the bias of Bi particles during the crystallization and solidification process [18,19]. With the increase of GNS content, the bias precipitation of Bi particles at the grain boundaries gradually increased, and holes started to appear in the tissue, as shown by the black circular dots in Figure 3b–f. The hole phenomenon worsened as more GNSs were added. During the casting process, due to the low density of GNSs, excess GNSs tended to float, increasing the possibility of creating holes.

### 3.2. Physical Properties

The melting characteristics parameters of the MC Sn-20Bi-*q*GNSs alloy are shown in Table 1. The melting point of the alloy prepared by the melt-casting method tends to decrease slightly overall with the addition of GNSs to the solder. The alloy melting point was lowest (191.4 °C) when the addition amount was 0.10 wt.%.

To study the effect of GNSs on the density of Sn-20Bi, the density of MC Sn-20Bi-*q*GNSs alloy was measured several times and averaged in this paper. Figure 2b shows that the addition of GNSs to the solder prepared by the melt-casting method significantly reduces its density by a maximum of about 11%. Because of the low density of GNSs, their uniform distribution in the matrix can reduce the density. The small amount of GNSs added does not have a significant effect on the theoretical density, so the actual density reduction is mainly due to the holes generated during the melt-casting process.

The wetting area and spread rate of MC Sn-20Bi-*q*GNSs composite solder on the copper substrate are shown in Figure 2c,d, respectively. According to the results, the wettability of the solder showed a decreasing and then increasing trend with the addition of GNSs at 0.10 wt.%. The excessive addition of GNSs led to an increase of holes in the solder, but the overall wettability of the alloy system was good.

### 3.3. Mechanical Properties

**Figure 4:**
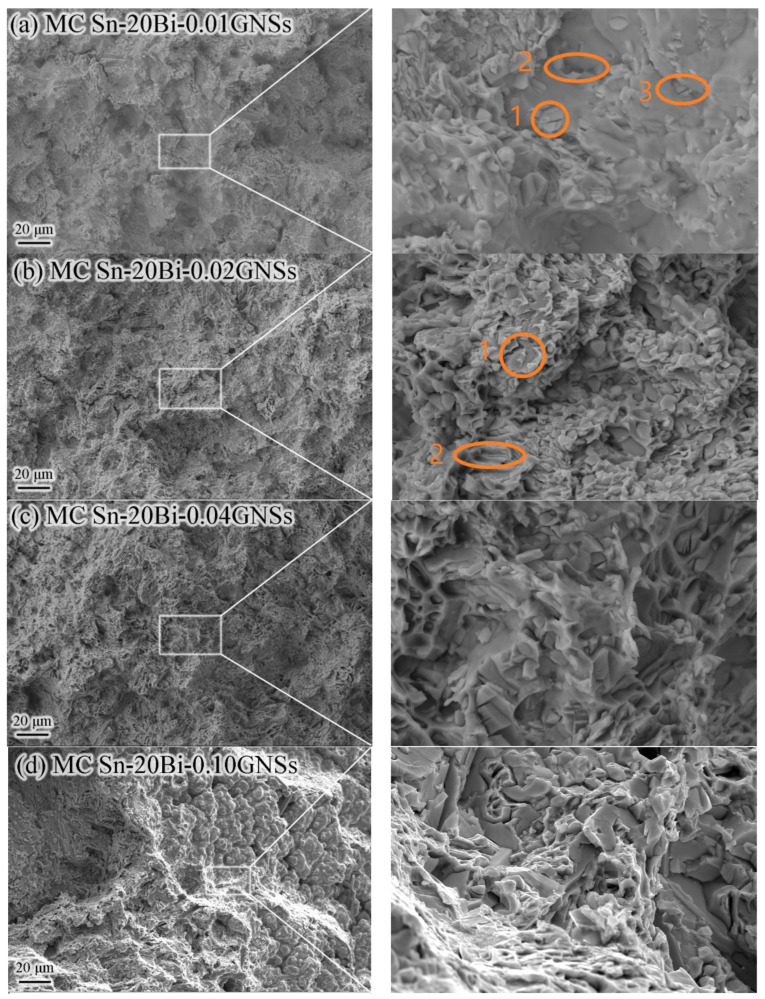
SEM images of the tensile fracture morphologies of MC Sn-20Bi-*q*GNSs alloys: (**a**) *q* = 0.01, (**b**) *q* =0.02, (**c**) *q* = 0.04, and (**d**) *q* = 0.10.

Tensile tests were performed on the composite solder to determine the mechanical properties of the solder. Figure 2e,f show the elongation and maximum tensile strength of the MC Sn-20Bi-*q*GNSs alloy, respectively. Additionally, Figure 4 shows the morphology of a typical alloy fracture. From Figure 2e, with the addition of GNSs, the alloy elongation shows an undulating trend of decreasing, then increasing, and then decreasing again. As can be seen in Figure 2f, the tensile strength of the composite solder increases substantially over the solder without the addition of GNSs, and the strengthening effect is obvious. The influence of GNSs on the elongation and tensile strength of the solder is mainly due to two factors. On the one hand, the presence of GNSs as a second phase in the Sn-20Bi solder matrix enhances the strength and reduces the plastic toughness. On the other hand, the addition of GNSs affects the mechanical properties of the solder by influencing the microstructure of the solder (e.g., the size of Bi particles, etc.). When 0.01 wt.% GNSs were added, the combination of increased Bi particles in the solder structure and second phase strengthening by GNSs decreased solder elongation (from 17.0% to 14.1%) and increased tensile strength (by 47.3%) to a maximum value of 99.6 MPa. Combined with the fracture morphology observed in Figure 4a, the fracture morphology of this alloy was found to have a small number of dimples and many grain penetration fracture morphologies. It is observed that some of the smaller particles of Bi in the solder debond from the matrix and form a small number of dimples, and the cracks break directly through the larger Bi particles.

Secondary cracks from coarse Bi particles that did not undergo debonding can be observed in circles 1 and 3 in Figure 4a. The inability of the coarse Bi particles to undergo debonding is the main reason for the decrease in the elongation of the alloy. Meanwhile, the Bi particles at the grain boundaries in circles 2 and 3 cause the extension of ductile cracks along the grain, leading to microporous aggregation. It is known from the Orowan mechanism that the fine Bi particles have a diffusive strengthening effect. When dislocation lines move under the action of external forces, the dislocation lines will be blocked by Bi particles, bend around the Bi particles, and finally pass by leaving a dislocation ring around the Bi particles. This bending of dislocation lines will increase the lattice distortion energy, which means increasing the resistance to dislocation motion and thus enhancing the strength. Therefore, when the particle spacing is smaller, the strengthening effect is more obvious, and the alloy strength increases.

When GNSs were added at 0.02 wt.%, GNSs refined the solder organization, resulting in a smaller and more uniformly distributed Bi particle size (Figure 3c). At this point, the effect of fine-grain strengthening enhanced the elongation of the composite solder (23.3%), which was 37% higher than that of Sn-20Bi. The maximum tensile strength of the composite solder was 97.3 MPa, which is consistent with the fracture morphology presenting a small portion as a deconvoluted fracture (circle 1 in Figure 4b) and a large portion as a tough nest fracture (circle 2 in Figure 4b).

At 0.04 wt.% and 0.06 wt.% of GNSs, the elongation, tensile strength, and fracture profile of the alloy were similar to those at 0.02 wt.%. When GNSs were added at 0.10 wt.%, the tensile strength of the alloy did not change much and remained at 89.0 Mpa, but the elongation dropped sharply to 9.7%. The fracture morphology also shows brittle fracture characteristics such as destructive facets and fractures along the crystal. This may be related to the porosity introduced by the floating of GNSs during the melting process and the enrichment of Bi particles at the grain boundaries.

### 3.4. Solder Joint Interface Microstructure

**Figure 5:**
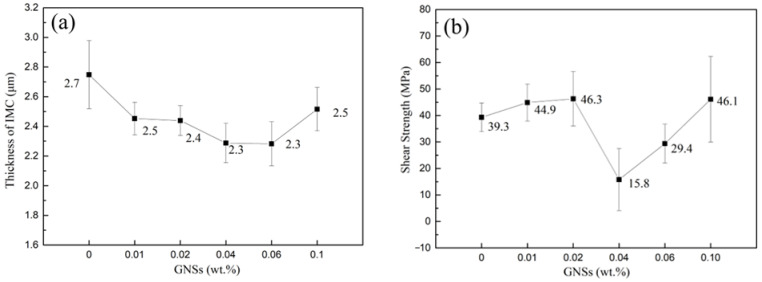
(**a**) Thickness of the IMC layer formed at MC Sn-20Bi-*q*GNSs/Cu and (**b**) shear strength of MC Sn-20Bi-*q*GNSs/Cu solder joint.

**Figure 6:**
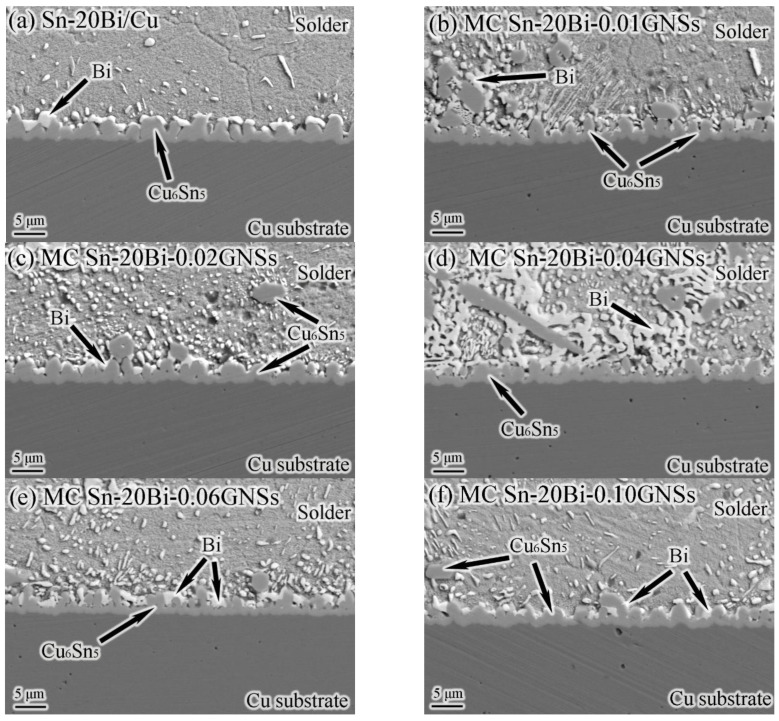
SEM images of MC Sn-20Bi-*q*GNSs/Cu solder joint cross section: (**a**) *q* = 0, (**b**) *q* = 0.01, (**c**) *q* = 0.02, (**d**) *q* = 0.04, (**e**) *q* = 0.06, and (**f**) *q* = 0.10.

The IMC thickness and microstructure of the MC Sn-20Bi-*q*GNSs/Cu solder joints after reflowing at 230 °C for 2 min are shown in Figure 5a and Figure 6, respectively. The microstructure of the solder consists of a gray Sn phase and a white granular/striped Bi phase. Sn-20Bi/Cu solder joints are enriched with Bi particles above the layer IMC at the interface. With the addition of GNSs, the Cu_6_Sn_5_ compound starts to appear in the matrix, while the Bi enriched at the interface starts to increase in the tissue. In particular, Bi phase segregation in the tissue increases and joins into lumps when the GNS content is 0.04 wt.%. With the addition of GNSs, the thickness of the IMC layer first decreases and then increases, as shown in Figure 6. This is because with the addition of GNSs, the bias aggregation of Bi particles at the IMC first increases and then decreases, and the fine Bi particles have a nailing effect at the IMC, which can inhibit the growth of IMC. Therefore, the variation law of IMC layer thickness is consistent with the enrichment law of Bi phase. At the same time, the low-density GNSs float to the IMC surface during reflow of the solder joints, acting as a barrier to atomic diffusion and grain boundary movement. The results show that the addition of GNSs has the effect of inhibiting the growth of IMC at the MC Sn-20Bi-*q*GNSs/Cu interface.

### 3.5. Shear Strength of Solder Joint

**Figure 7:**
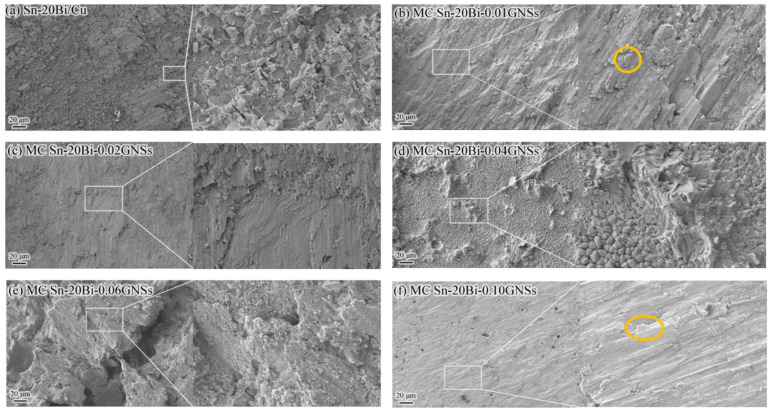
SEM images of MC Sn-20Bi-*q*GNSs/Cu solder joint after shear fracture: (**a**) *q* = 0, (**b**) *q* = 0.01, (**c**) *q* = 0.02, (**d**) *q* = 0.04, (**e**) *q* = 0.06, and (**f**) *q* = 0.10.

The shear strength and shear fracture morphology of the MC Sn-20Bi-*q*GNSs/Cu solder joints are shown in Figure 5b and Figure 7, respectively. Figure 5b shows that with the addition of GNSs, the shear strength of the alloyed joints increases then decreases, and then increases again. When the GNS content was less than 0.02 wt.%, the shear strength of the joints increased as the GNS content increased. The shear strength was 46.3 MPa at 0.02 wt.% GNS content, which was 17.8% higher than that without the addition of GNSs. However, the shear strength was reduced to 15.8 MPa when the GNS content was 0.04 wt.%. In conjunction with Figure 6d, the alloy is enriched with a large number of brittle phase Bi particles at the interface of the welded joints, making the alloy welded joints prone to fracture at the interface, as shown in the fracture morphology in Figure 7d. After that, the shear strength of the solder joint increases gradually with the increase of GNS content until it reaches 46.1 MPa.

The shear fracture morphology of the solder junctions without and with the addition of 0.04 wt.% GNSs is a visible brittle fracture along the crystal whose fracture site occurs inside the IMC and at the solder/IMC interface, respectively, as shown in Figure 7a,d. The fracture site of the other alloyed solder joints occurred inside the solder, as shown in Figure 7b–f. Obvious slip bands and slip steps can be observed in the fracture morphology, and a large amount of slip deformation (plastic deformation) is a characteristic of ductile fracture. Meanwhile, a small amount of decoupled tongue-like fracture morphology can be observed in Figure 7b,f (orange circles). In summary, the optimal shear strength of this alloy system is MC Sn-20Bi-0.02GNSs/Cu solder joints.

### 3.6. Corrosion Rate Analysis

**Figure 8:**
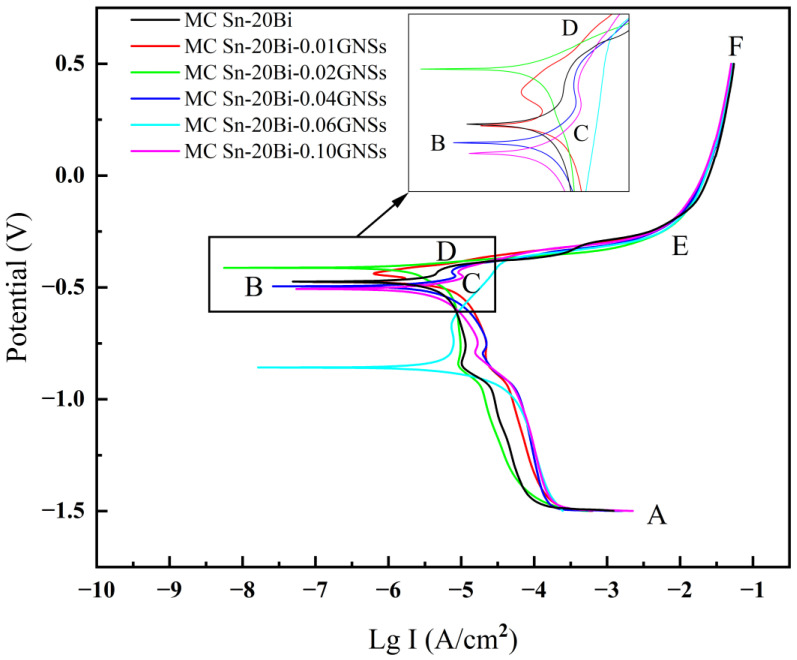
Polarization curves of MC Sn-20Bi-*q*GNSs alloys in 3.5 wt.% NaCl solution.

The dynamic potential polarization curves of MC Sn-20Bi-*q*GNSs (*q* = 0, 0.01, 0.02, 0.04, 0.06, 0.10 wt.%) composite solder in 3.5 wt.% NaCl solution is shown in Figure 8. Based on the principle of extrapolation, the electrochemical corrosion parameters of Table 2 were obtained. With the addition of GNSs, the corrosion current density and corrosion rate of the composite solder showed the law of first decreasing and then increasing. The corrosion current density and corrosion rate were 0.17 × 10^−6^ A/cm^2^ and 0.005 mm/year, respectively, when GNSs were added at 0.01 wt.%. The corrosion potential was also higher than that of the original sample without GNSs, indicating that the Sn-20Bi-0.01GNSs composite solder had the best corrosion resistance.

The corrosion process of the alloy in NaCl solution and its reactions can be analyzed by the polarization curve. The AB segment is the cathodic region of an electrochemical reaction in which oxygen absorption and hydrogen precipitation reactions occur in turn [20,21].

(1) $$O_{2}+4e^{-}+2H_{2}O\to 4{\mathrm{OH}}^{-}$$

(2) $$2H_{2}O+2e^{-}\to H_{2}+2{\mathrm{OH}}^{-}$$

Point B is the junction point of the cathodic reaction and anodic reaction, corresponding to the potential for corrosion potential (E_corr_). The value of the corrosion current density (i_corr_) is determined by means of a Tafel graph. The i_corr_ value is obtained by extrapolating the points where the cathode and anode Tafel slopes intersect each other. The corrosion rate of the material depends on the i_corr_ value. The lower the i_corr_ value, the lower the corrosion rate of the material [22]. With increasing current density, the solder begins to dissolve tin reaction, and the surface is gradually corroded.

(3) $$\mathrm{Sn}\to {\mathrm{Sn}}^{2+}+2e^{-}$$

(4) $$\mathrm{Sn}\to {\mathrm{Sn}}^{4+}+4e^{-}$$

In the meantime, the Sn ions combine with OH- in the water to produce a hydroxide of Sn, i.e., Sn (II), Sn (IV), Sn (OH)_2_, and Sn (OH)_4_.

(5) $${\mathrm{Sn}}^{2+}+2{\mathrm{OH}}^{-}\to \mathrm{Sn}{\left(\mathrm{OH}\right)}_{2}$$

(6) $${\mathrm{Sn}}^{4+}+4{\mathrm{OH}}^{-}\to \mathrm{Sn}{\left(\mathrm{OH}\right)}_{4}$$

Sn(OH)_2_ and Sn(OH)_4_ dehydrate and form SnO and SnO_2_.

(7) $$\mathrm{Sn}{\left(\mathrm{OH}\right)}_{2}\to \mathrm{SnO}+H_{2}O$$

(8) $$\mathrm{Sn}{\left(\mathrm{OH}\right)}_{4}\to {\mathrm{SnO}}_{2}+2H_{2}O$$

In the CD region, the current is not influenced by the potential, and this region is called the stable passivation phase. As can be seen from the polarization curves, the addition of GNSs in amounts less than 0.02 wt.% leads to a shift of the corrosion potential to better values and a decrease in the corrosion current density. Correspondingly, the lower the value of i_corr_, the lower the corrosion rate of the Sn-20Bi-GNSs solder alloy. The addition of trace amounts of GNSs in the solder acts as both fine grain strengthening and diffusion strengthening. This causes a reduction in the grain size of Sn as an anode for galvanic corrosion as well as an increase in grain boundaries, which has a hindering effect on continuous corrosion. Thus, the corrosion rate is reduced. Moreover, GNSs have strong corrosion resistance in the solder matrix, which can effectively hinder the occurrence of corrosion [19].

Starting from point D, the current density increases sharply and continuously because the passivation film is broken down. In addition, at point E, the current density starts to decrease continuously. Finally, due to the occurrence of a pseudopassivation process and the perceived formation of a corrosion film, the current remains constant as the potential increases.

### 3.7. Analysis of Corrosion Products

**Figure 9:**
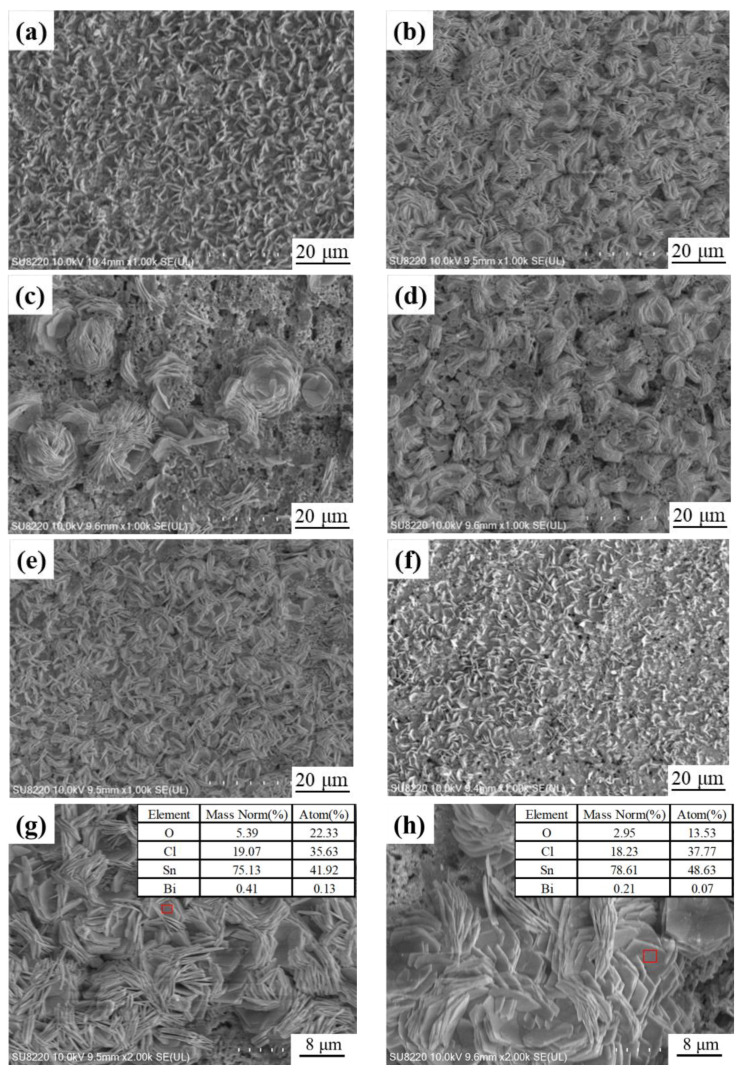
SEM images of corrosion morphology; (**a**–**f**) the morphologies of MC Sn-20Bi-*q*GNSs (*q* = 0, 0.01, 0.02, 0.04, 0.06, 0.1wt.%); (**g**,**h**) EDS analysis results of MC Sn-20Bi-0.01GNSs and MC Sn-20Bi-0.02GNSs corrosion products (red frames).

**Figure 10:**
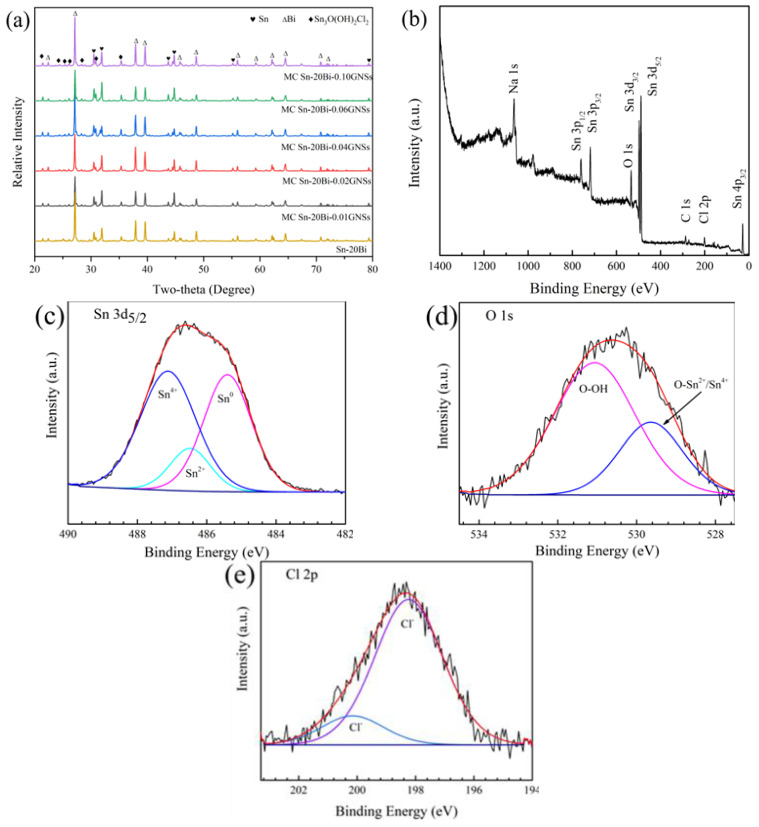
XRD patterns of MC Sn-20Bi-*q*GNSs solders after electrochemical corrosion; (**b**) XPS scan full spectrum of MC Sn-20Bi-0.02GNSs solder after electrochemical corrosion; (**c**–**e**) XPS fitting results of MC Sn-20Bi-0.02GNSs after electrochemical corrosion: (**c**) Sn 3d5/2, (**d**) O 1s, and (**e**) Cl 2p.

At the end of the process, SEM (Figure 9a–f) and EDS analyses (Figure 9g,h red frames) were performed on the surface of the corroded samples to study the morphology of the corrosion products, and XRD (Figure 10a) was performed to study the corrosion products. SEM micrographs showed the presence of sheet-like corrosion products on the surface of the alloys to which GNSs were added. The presence of lamellar corrosion products provides various cavities. These cavities are areas where Cl ions are easily targeted and eventually lead to an increase in the corrosion rate of the alloy. Under the scanning electron microscope, it was found that the MC Sn-20Bi-0.02GNSs alloy had large uneven lamellar agglomerates on its surface, and many holes could be seen on the surface. The MC Sn-20Bi and Sn-20Bi-0.1GNSs alloys had very fine lamellar distribution on their surfaces, exposing a large area of alloy. The MC Sn-20Bi-0.01GNSs and MC Sn-20Bi-0.06GNSs have a more uniform distribution of flakes on the surface and expose a smaller area of alloy. Based on EDS and XRD analysis, it can be assumed that the flakes on the surface are oxides after corrosion. The exposed alloy below the oxide forms a circuit with good electrical conductivity, allowing corrosion to continue, thus making the corrosion resistance less. The corrosion products that are uniformly distributed on the surface of the alloy and have good densities have a certain protective function for the alloy and can prevent further corrosion from occurring.

To determine the corrosion reaction, XPS was used to analyze the solder corrosion products, and the results are shown in Figure 10b–e The results show that the peaks of Sn 3d, Sn 3p, Sn 4p, O 1s, Cl 2p, Na 1s, and C 1s are present in the full spectrum of Figure 10b. Figure 10c–e shows the split peak fits of the narrow sweep peaks of Sn 3d5/2, O 1s, and Cl 2p. The results showed that the Sn 3d_5/2_ spectrum contains three fitted peaks of 487.1 eV (Sn^4+^), 486.5 eV (Sn^2+^), and 485.4 eV (Sn^0^); the binding energies of the two fitted peaks of O 1s are 531.1 eV and 529.6 eV, respectively, corresponding to hydroxide and oxygen in oxide [23]; the Cl 2p spectra were fitted by two peaks of 200.2 eV and 198.2 eV, which correspond to the Na-Cl and Sn-Cl compounds, respectively.

## 4. Conclusions

In this work, the MC Sn-20Bi-qGNSs composite solder was prepared by the melt-casting method. The effect of GNSs on the organization, physical properties, mechanical properties, and corrosion resistance of the composite solder was investigated. The following conclusions can be drawn.

1. With the addition of GNSs, solder melting point tends to decrease. The generation of holes during the melt-casting process results in a decrease in the density and wettability of the composite solder.
2. The addition of GNSs enhances the tensile strength of MC Sn-20Bi composite solder up to 99.6 MPa. However, with the addition of GNSs, the tensile strength and elongation show a decreasing trend. This may be related to the holes caused by the uplift of GNSs during the melting process and the enrichment of Bi particles at the grain boundaries.
3. GNSs can act as a barrier to atomic diffusion and grain boundary movement to inhibit the growth of IMC at the MC Sn-20Bi-*q*GNSs/Cu interface. The addition of 0.02 wt.% GNSs was able to maximize the shear strength of MC Sn-20Bi solder joints up to 46.3 MPa.
4. The corrosion product of MC Sn-20Bi-*q*GNSs in 3.5% NaCl was Sn_3_O(OH)_2_Cl_2_, and the composite solder had the best corrosion resistance when the GNSs content was 0.01 wt.%.

## Figures and Tables

**Table 1:** DSC analysis results of MC Sn-20Bi-*q*GNSs alloys (Temperature: °C).

Alloy	Start 1	Peak1	End 1	Start 2	Peak2	End 2	Melting Range
Sn-20Bi	137.7	139.4	140.9	187.0	201.7	210.0	23.0
MC Sn-20Bi-0.01GNSs	138.4	140.0	141.4	176.1	194.6	202.2	26.1
MC Sn-20Bi-0.02GNSs	138.2	139.6	140.8	179.8	195.9	202.3	22.5
MC Sn-20Bi-0.04GNSs	137.7	139.8	141.8	177.6	195.0	201.8	24.25
MC Sn-20Bi-0.06GNSs	138.2	139.5	141.4	176.6	193.2	199.6	23.1
MC Sn-20Bi-0.10GNSs	138.5	139.7	141.2	173.9	191.4	197.5	23.6

**Table 2:** Electrochemical corrosion parameters of MC Sn-20Bi-*q*GNSs alloys in 3.5 wt.% NaCl solution.

Alloy	i_corr_ (A/cm^2^)	E_corr_ (V)	Corrosion Rate (mm/year)
MC Sn-20Bi	7.97 × 10^−6^	−0.475	0.211
MC Sn-20Bi-0.01GNSs	0.17 × 10^−6^	−0.444	0.005
MC Sn-20Bi-0.02GNSs	2.11 × 10^−6^	−0.414	0.056
MC Sn-20Bi-0.04GNSs	10.80 × 10^−6^	−0.497	0.286
MC Sn-20Bi-0.06GNSs	11.60 × 10^−6^	−0.859	0.308
MC Sn-20Bi-0.1GNSs	15.70 × 10^−6^	−0.508	0.416

## Data Availability

The datasets generated during and analyzed during the current study are available from the corresponding author on reasonable request.
